# Supplementary material for: Rate volatility and asymmetric segregation diversify mutation burden in cells with mutator alleles
Source: Commun Biol. 2021 Jan 4;4:21. doi: 10.1038/s42003-020-01544-6 (PMC7782790; doi:10.1038/s42003-020-01544-6)
Supplement: Supplementary file 2 — Supplementary Information [file 42003_2020_1544_MOESM2_ESM.pdf]

Supplementary Information

Corresponding author: Alan J. Herr, alanherr@uw.edu

Haploid *pol2-4 msh6Δ* Mutators

Prior to switching to stronger diploid mutators, we first obtained full replication error counts for 44 *pol2-4 msh6Δ* divisions from 7 independent lineages, encompassing 308 mutations (Supplementary Table 1, Supplementary Data 2). We sequenced only those clones that would contribute to a full error count (Supplementary Fig.1). Since our previous study suggested that mutations arose in *pol2-4 msh6Δ* mother cells at a rate of 0.4 or 4 mutations/genome/division[1], with full replication error counts, the volatility model predicts two well-separated Poisson distributions centered around 1.6 and 16 replication errors per division. Instead, we observed a single distribution centered around 6.5 (±3.9) replication errors per division. The distribution of full replication error counts in *pol2-4 msh6Δ* cells had a $\hat{D}$ of 2.2, which is consistent with a less pronounced volatility of the *pol2-4 msh6Δ* mutator phenotype. In keeping with this interpretation, fitting these data to different probability distributions revealed they matched a negative binomial better than a single or two-Poisson mixture as judged by AIC. Parsing this data into the number of mutations per individual cell division produces a distribution (N=176) that fits a single Poisson with a rate of 1.75 mutations/division. This finding does not negate the hypothesis of a mild mutator volatility based on the full replication error counts. The expected dispersions of mutations in *pol2-4 msh6Δ* haploid cells (n=176, λ=1.75) were comparable for the Poisson-binomial ($\hat{D}$ = 1.13±0.12) and Poisson ($\hat{D}$ = 1.0±0.1) models (Supplementary Fig. 6). The rate of 1.75 mutations/division lies almost directly between the predicted underlying rates from our published two-Poisson Model[1]. Thus, our previous distribution likely contained a preponderance of cell divisions with this intermediate mutation rate. The high number of divisions in that earlier dataset with no mutations could have partly been the result of a biological "zero-inflation" due to the unequal sharing of mutations described in Fig. 3 for *pol3-01/pol3-01 msh6Δ/msh6Δ* cells. If so, why are there fewer cells with 0 mutations in the current distribution? We suspect that the stringent requirement of eight viable clones to obtain a full replication error count may have introduced an ascertainment bias. Due to unequal sharing of mutations, members of the lineage with the highest number of mutations may fail to form a colony. The reciprocal clones with no errors from that same division would also not be scored. This potential ascertainment bias would affect our estimates of mutator volatility, since divisions with a higher mutation rate are more likely to have at least one progeny fail to form a colony.

Colonies Not Included In Analysis

Many complete sub-lineages (comprised of d, gd1, gd2, and ggd) were not sequenced because inviability later in the lineage prevented us from gaining a full replication error count. For instance, full replication error counts for AH120 divisions that yielded d3 and d4 are not possible because the d5 sublineage was completely inviable (Supplementary Fig. 1). Likewise, sometimes colonies within informative sub-lineages (e.g. AH119 gd8-2, AH120 gd10-2/ggd10, AH121 gd9-1) were not sequenced because they were not required for a full replication error count. In some cases (AH156 d14, AH160 d5, AH157 d8), colonies that provided identical information on a division segregant subgroup were sequenced when the preferred colony failed to form a viable clone (Supplementary Fig.2). In other cases (AH158 gd4-2/ggd4, AH158 gd7-2/ggd7, AH162 gd6-2/ggd6), colonies had poor sequencing coverage and were censored from

the analysis. In rare cases, sequence analysis and review of dissection notes suggested an assignment error occurred; however, our careful analysis of the patterns of shared mutations enabled post-hoc deconvolution of the events. In one example, in the 8th division of AH156, we separated 3 cells from the mother. The two larger, similarly sized cells were both clearly daughter cells and the smaller cell was a granddaughter cell, although it was not clear which was its parent. We moved the daughter cells to the d8 (AH15629) and d9 positions (AH15633) and placed the smaller cell below in the gd8-1 position (AH15630). We then proceeded to isolate the remaining members of the lineage. Sequencing analysis of the resulting colonies revealed that AH15633 was in fact d8, AH15629 was d9, and AH15630, the daughter of AH15633, not AH15729. This error meant we had to reorder the lineage. AH15631 was gd9-1 not gd8-2. AH15632, daughter of AH15630, was ggd8 and AH15634 was gd8-2. Since we didn't realize AH15631 was gd9-1, we failed to dissect her first daughter to serve as ggd9 and consequently were unable to obtain a full replication error count for the 9th division. In another example, AH160 division 2 was censored entirely from analysis after finding from the pattern of mutations that AH16005 was a granddaughter derived from d1 of this lineage (AH16001).

Distribution of Mutations and Spectrum

Plotting the mutations scored from all divisions of *pol3-01/pol3-01 msh6Δ/msh6Δ* mutator mother cells reveals mutations were generated across much of the unmasked portions of the sequenced genome (Supplementary Fig.3a). Upon investigation, the few tracts of unmasked chromosomes lacking mutations are likely artifacts of regions of low sequence coverage which consistently fell below our target thresholds for quality and depth in at least one or more members of a lineage.

As expected, C→T mutations are the most abundant single nucleotide substitutions, followed by T→C and C→A. The trinucleotide context reveals a prominent peak of C→A mutations at a TCT context, a hallmark of proofreading deficiency[2, 3], as well as a peak at CCT (Supplementary Fig. 3b).

Supplementary Table 1

Lineage[a]	Scored Sites[b]	Mutations[c]	Divisions[d]	Mutation Rate[e]
pol2-4 msh6				
119	11,080,506	33	5	0.006
120	11,120,599	51	7	0.0066
121	11,203,731	38	6	0.0057
122	10,909,400	59	7	0.0077
123	11,099,548	24	4	0.0054
124	10,094,023	58	10	0.0057
125	10,897,295	45	5	0.0083
Total		*308*	*44*	
Mean (stdev ±)	10,915,014 (3.51 x 10^5)	44 (12)	6.3 (1.8)	0.0065 (0.001)
pol3-01/pol3-01 msh6Δ/msh6Δ				
151	10,541,044	1,597	6	2.53
153	10,636,360	1,582	6	2.48
156	9,521,963	2,976	10	3.13
157	10,594,546	1,599	6	2.52
158	9,818,623	1,347	5	2.74
160	9,679,326	1,543	5	3.19
162	8,578,576	3,157	12	3.07
Total		*13,801*	*50*	
Mean (stdev ±)	9,910,063 (6.95 x 10^5)	1,972 (699)	7 (2.5)	2.81 (0.29)

[a] Lineage refers to descendants of the same mother cell. See Supplementary Figs. 1 and 2 for images of colonies and Supplementary Data 1 and 2 for mutations.

[b] Scored sites refers to the number of genomic nucleotide positions confidently scored in all members of the lineage.

[c] The total number of independent mutations identified within each lineage.

[d] The number of divisions with full replication error counts (see Fig. 1).

[e] Mutation rate (x 10^{-5} mutations/bp/division): the number of mutations divided by the total number scored sites divided by the number of divisions.

Supplementary Figures

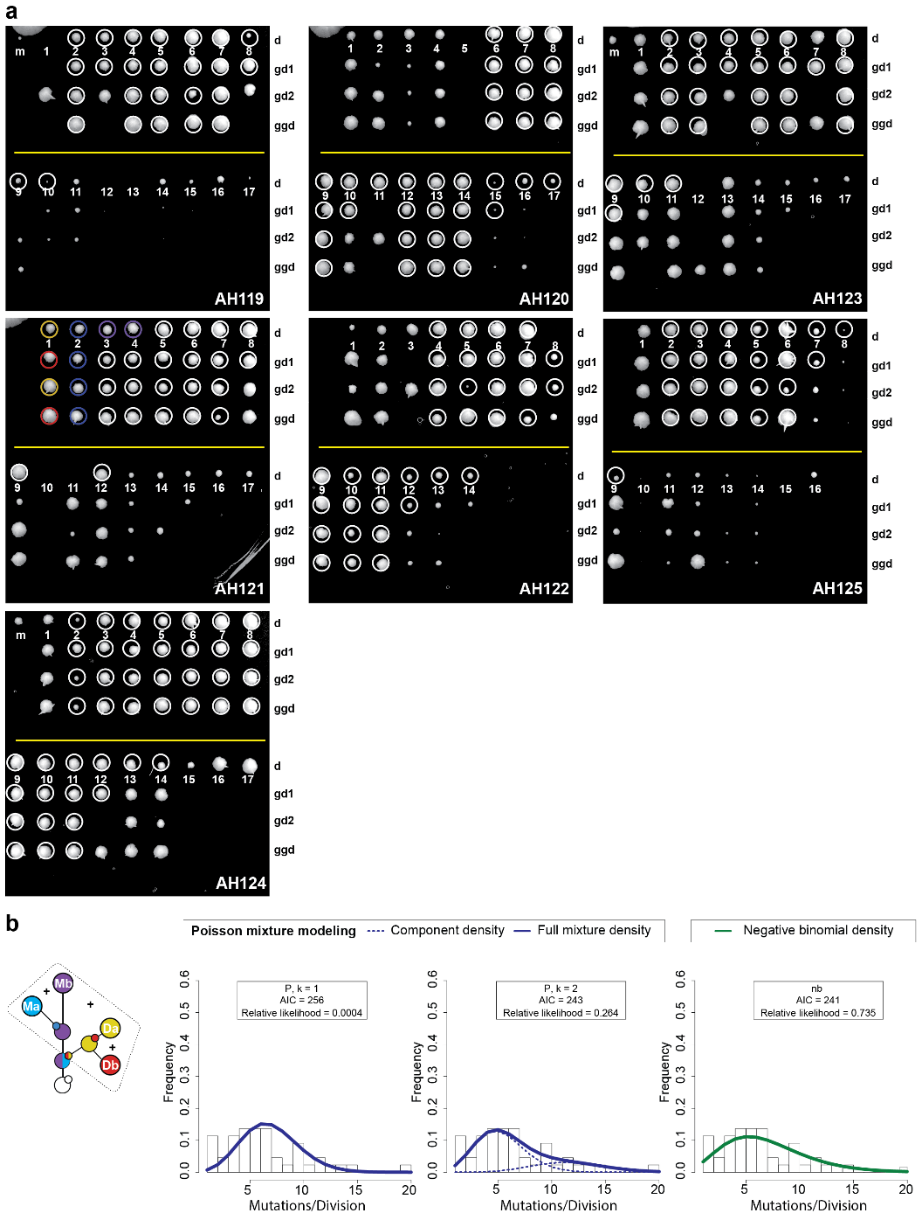

Supplementary Fig. 1: *pol2-4 msh6Δ* lineages.

a, Photographs of agar plates with colonies formed from single cell lineages. The lineage number is given in the lower right-hand corner. Locations of rows of daughter (d), first granddaughter (gd1), second granddaughter (gd2), and great-granddaughter (ggd) colonies are given on the right-hand side of the images. Sublineage number is indicated below each daughter colony. Circles indicate sequenced colonies. Colored circles in Lineage AH121 illustrate segregant groupings for the first division (Fig. 1). Yellow line divides earlier sublineages from later sublineages. Gaps in colony growth reflect lethality. **b**, Fitting the distributions of full error counts from haploid *pol2-4 msh6Δ* divisions (*n* = 44) to alternative models. k=1, single Poisson; k=2, two-Poisson; nb, negative binomial; AIC, Akaike information criterion.

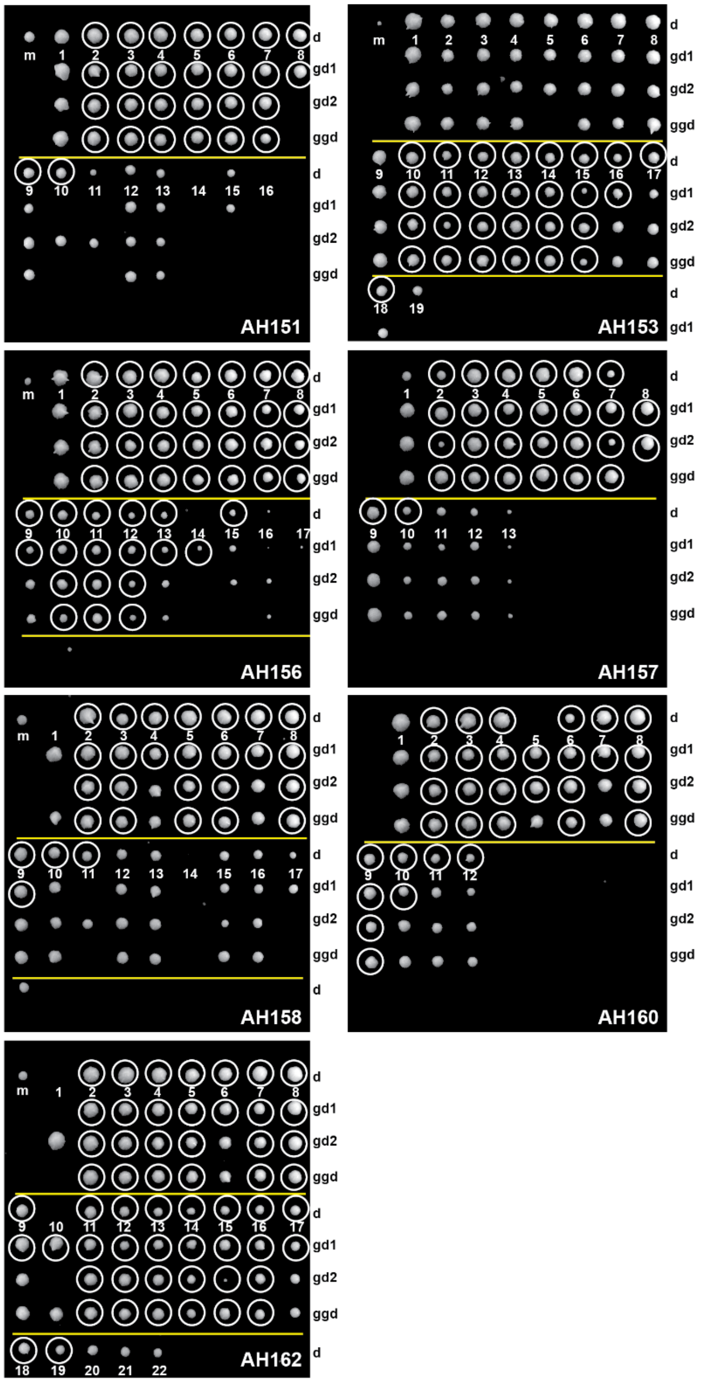

Supplementary Fig. 2: ***pol3-01/pol3-01 msh6Δ/msh6Δ*** **lineages.**

Photographs of agar plates with colonies formed from single cell lineages. The lineage number is given in the lower right-hand corner. Locations of rows of daughter (d), first granddaughter (gd1), second granddaughter (gd2), and great-granddaughter (ggd) colonies are given on the right-hand side of the images. Sublineage number is indicated below each daughter colony. Yellow line divides earlier sublineages from later sublineages. Gaps in colony growth reflect lethality.

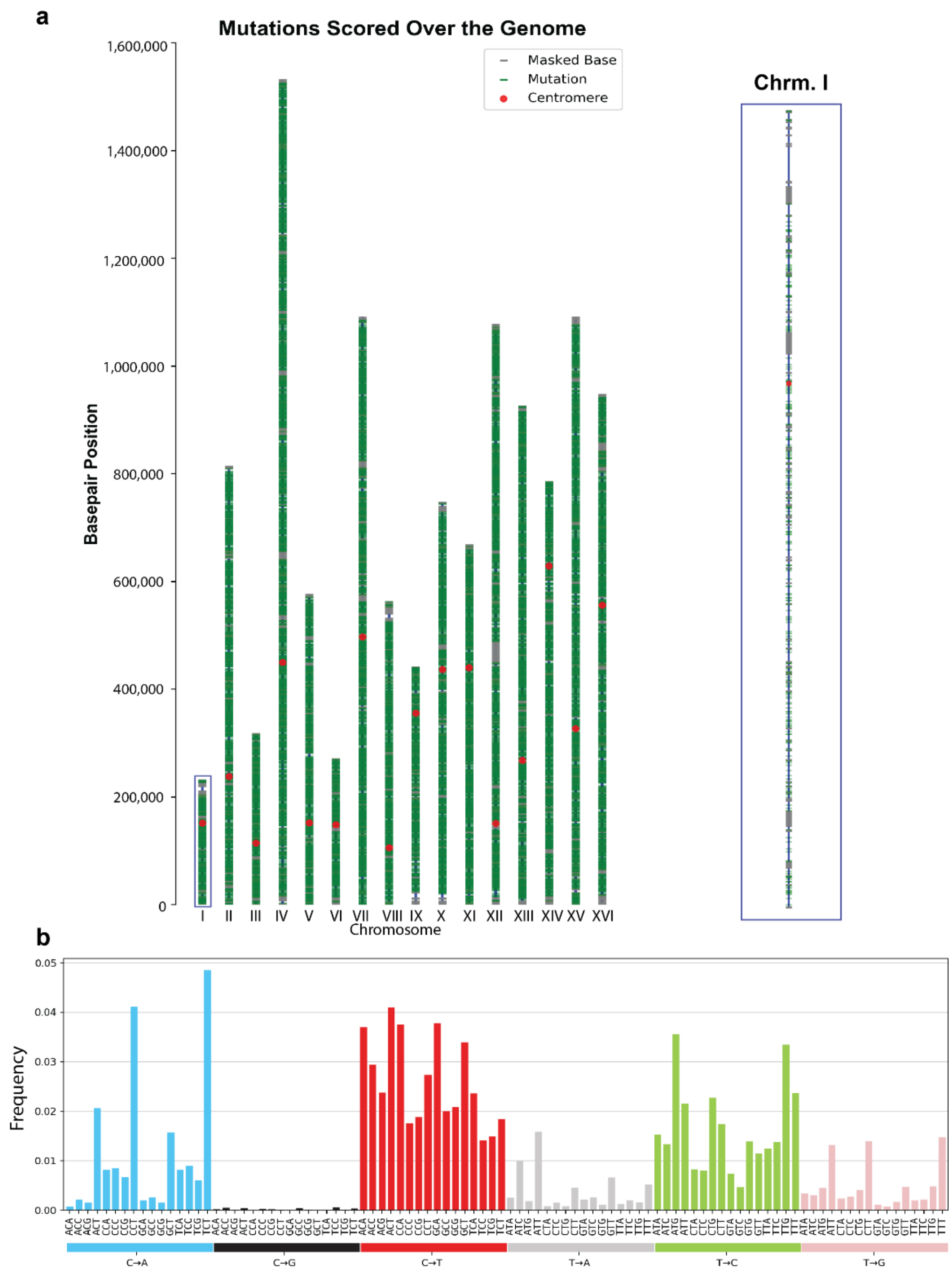

Supplementary Fig. 3: Distribution of mutations and spectra in *pol3-01/pol3-01 msh6Δ/msh6Δ* lineages.

a, Genome level distribution of mutations (green) over yeast chromosomes (blue lines). 13,801 mutations pooled from 50 scored divisions of *pol3-01/pol3-01 msh6Δ/msh6Δ* diploid mother cells, representing approximately 1 mutation per 1000 bases of the yeast genome. Close up view of representative chromosome I (right). Masked bases are represented by grey ticks. **b**, 96-trinucleoide mutation spectra context of all mutations (spectrum) by frequency that arose over 50 divisions of *pol3-01/pol3-01 msh6Δ/msh6Δ* diploid mother cells, generated using the snv-spectrum program (https://github.com/aroth85/snv-spectrum).

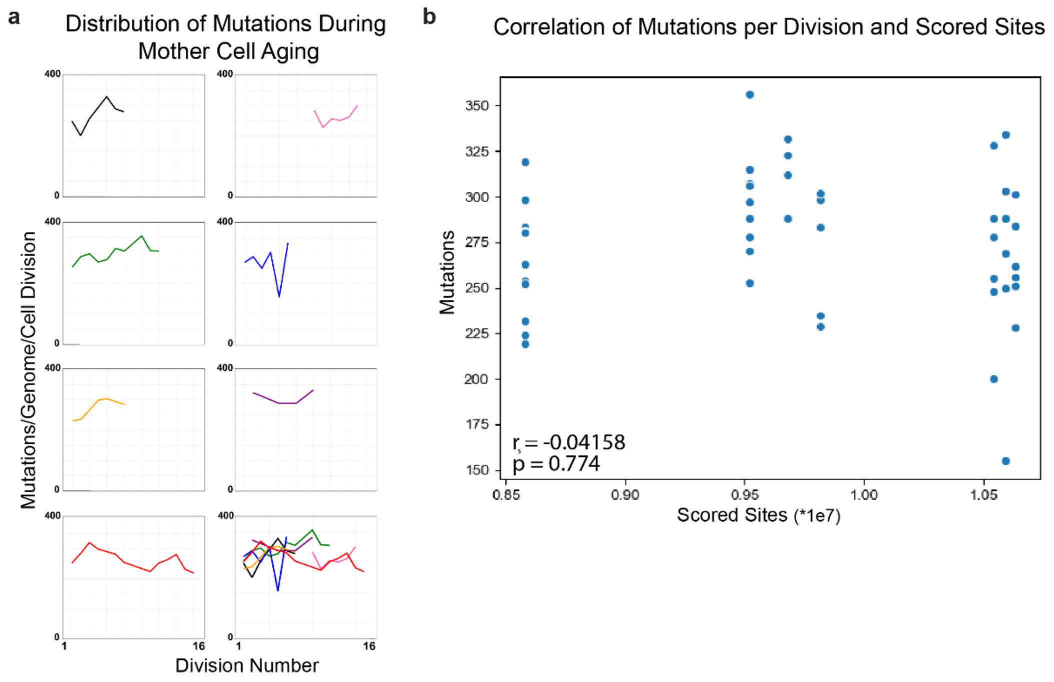

Supplementary Fig. 4: Excluding simple explanations for *pol3-01/pol3-01 msh6Δ/msh6Δ* mutator volatility.

a, Mutation counts and maternal age. The total mutation counts from individual divisions is plotted relative to maternal age (Division number). **b**, Mutation counts and size of scored genome. The proportion of the genome scored in all members of a lineage varies between lineages due to sequencing depth and number of lineage members, but is not correlated with mutation counts (Spearman Correlation).

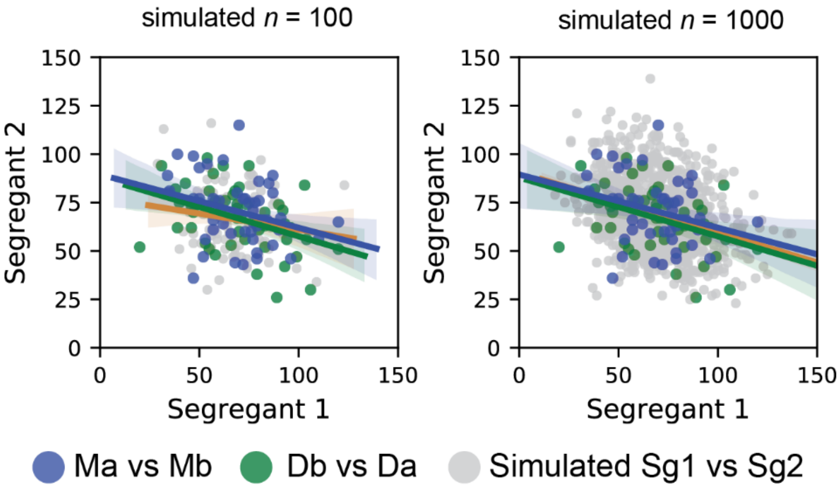

Supplementary Fig. 5: Correlations between segregation groups.

X/Y Scatter plots of segregant group pairs (Db/Da, Ma/Mb, *n* = 50 for each) from *pol3-01/pol3-01 msh6Δ/msh6Δ* divisions are plotted alongside simulated data (*n* = 100, left; *n* = 1000, right). Segregant 1 corresponds to Db or Ma (daughters). Segregant 2 corresponds to Da or Mb (mothers). The furthest outlier point for Ma/Mb (65,120) in the upper-half of the plot comes from Division 8 (Supplementary Data 1), which produced a Db/Da point located at (120,51; on the green line) that yielded similar mismatch totals (Dm, 171; Mm, 185). This suggests the division had a high mutation rate. Ma/Mb (47,36) and Db/Da (20, 52) segregant pairs in the lower left-hand quadrant also appear as outliers. Both pairs are derived from Division 15 (Supplementary Data 1), leading to the conclusion that the mutation rate in that division was inherently low.

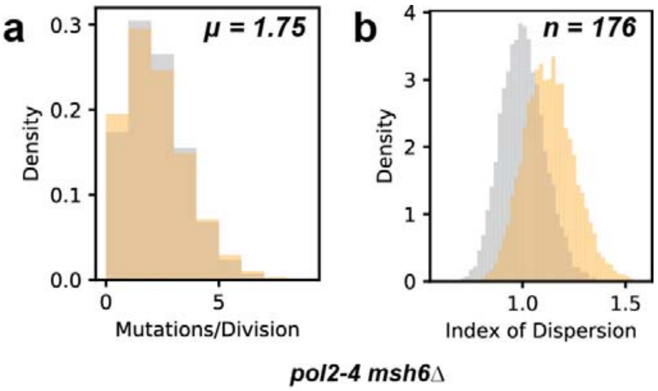

Supplementary Fig. 6: Simulation of *pol2-4 msh6Δ* haploid mutagenesis

a, The simulated distribution of mutations from haploid *pol2-4* m*sh6Δ* cells at a rate of μ = 1.75 mutations/division (*n* = 10,000) assuming a single Poisson process (grey) or a Poisson-binomial process (orange). **b**, Variation in the index of dispersion of simulated data from Poisson and Poisson-binomial models (*n* = 176) over 10,000 iterations.

Supplementary References

1. Kennedy SR, Schultz EM, Chappell TM, Kohrn B, Knowels GM, Herr AJ. Volatility of Mutator Phenotypes at Single Cell Resolution. *PLoS Genet.* **11**, e1005151 (2015).

2. Alexandrov LB*, et al.* Signatures of mutational processes in human cancer. *Nature* **500**, 415-421 (2013).

3. Shinbrot E*, et al.* Exonuclease mutations In DNA polymerase epsilon reveal replication strand specific mutation patterns and human origins of replication. *Genome Res.*, (2014).
