# Supplementary material for: Rate volatility and asymmetric segregation diversify mutation burden in cells with mutator alleles
Source: Commun Biol. 2021 Jan 4;4:21. doi: 10.1038/s42003-020-01544-6 (PMC7782790; doi:10.1038/s42003-020-01544-6)
Supplement: Supplementary file 3 — Description of Additional Supplementary Files [file 42003_2020_1544_MOESM3_ESM.pdf]

Description of Additional Supplementary Files

File Name: Supplementary Data 1
Description:

***pol3-01/pol3-01 msh6Δ/msh6Δ* sequencing data**

Page	*Description*
SRA submission	List of sequence files submitted to the Short Read Archive (SRA)
Mutation Summary	Summary of mutation counts from different single cell lineages
MutInSegGroups	Table of mutation counts broken down into segregant groups
MutInSegGroupsChr	Segregant group table further broken down by chromosome
Full Mutation List	All mutations observed, organized by segregant groups.
Lineage 151	Sorted spreadsheet from Lineage 151 showing variants in segregant groups
Lineage 153	Sorted spreadsheet from Lineage 153 showing variants in segregant groups
Lineage 156	Sorted spreadsheet from Lineage 156 showing variants in segregant groups
Lineage 157	Sorted spreadsheet from Lineage 157 showing variants in segregant groups
Lineage 158	Sorted spreadsheet from Lineage 158 showing variants in segregant groups
Lineage 160	Sorted spreadsheet from Lineage 160 showing variants in segregant groups
Lineage 162	Sorted spreadsheet from Lineage 162 showing variants in segregant groups

File Name: Supplementary Data 2
Description:

***pol2-4 msh6Δ* sequencing data**

Page	*Description*
SRA submission	List of sequence files submitted to the Short Read Archive (SRA)
Mutation Summary	Summary of mutation counts from different single cell lineages
Full Mutation List	All mutations observed, organized by segregant groups.
R1_Lineage	Sorted spreadsheet from Lineage R1 showing variants in segregant groups
R2_Lineage	Sorted spreadsheet from Lineage R2 showing variants in segregant groups
R4_Lineage	Sorted spreadsheet from Lineage R4 showing variants in segregant groups
R5_Lineage	Sorted spreadsheet from Lineage R5 showing variants in segregant groups
R6_Lineage	Sorted spreadsheet from Lineage R6 showing variants in segregant groups
R9_Lineage	Sorted spreadsheet from Lineage R9 showing variants in segregant groups
R10_Lineage	Sorted spreadsheet from Lineage R10 showing variants in segregant groups
